# Microglia become hypofunctional and release metalloproteases and tau seeds when phagocytosing live neurons with P301S tau aggregates

**DOI:** 10.1126/sciadv.abg4980

**Published:** 2021-10-20

**Authors:** Jack H. Brelstaff, Matthew Mason, Taxiarchis Katsinelos, William A. McEwan, Bernardino Ghetti, Aviva M. Tolkovsky, Maria Grazia Spillantini

**Affiliations:** 1 Department of Clinical Neurosciences, Clifford Allbutt Building, University of Cambridge, Cambridge CB2 0AH, UK.; 2 UK Dementia Research Institute Cambridge, Island Research Building, University of Cambridge, Cambridge CB2 0AH, UK.; 3 Department of Pathology and Laboratory Medicine, Indiana University, Indianapolis, IN, USA.

## Abstract

The microtubule-associated protein tau aggregates in multiple neurodegenerative diseases, causing inflammation and changing the inflammatory signature of microglia by unknown mechanisms. We have shown that microglia phagocytose live neurons containing tau aggregates cultured from P301S tau mice due to neuronal tau aggregate-induced exposure of the “eat me” signal phosphatidylserine. Here, we show that after phagocytosing tau aggregate-bearing neurons, microglia become hypophagocytic while releasing seed-competent insoluble tau aggregates. These microglia express a senescence-like phenotype, demonstrated by acidic β-galactosidase activity, secretion of paracrine senescence-associated cytokines, and maturation of matrix remodeling enzymes, results that are corroborated in P301S mouse brains and ex vivo brain slices. In particular, the nuclear factor κB–dependent activation of matrix metalloprotease 3 (MMP3/stromelysin1) was replicated in brains from patients with tauopathy. These data show that microglia that have been activated to ingest live tau aggregates-bearing neurons behave hormetically, becoming hypofunctional while acting as vectors of tau aggregate spreading.

## Teaser

Microglia change their behavior when eating live neurons containing tau protein aggregates, a hallmark of Alzheimer’s disease.

## INTRODUCTION

Aggregation of the protein tau from a soluble unfolded state to an insoluble, β sheet–enriched filamentous structure underlies numerous human neurodegenerative diseases known as tauopathies (*1*). These include Alzheimer’s disease (AD), various frontotemporal dementias, Pick’s disease, progressive supranuclear palsy (PSP), corticobasal degeneration, chronic traumatic encephalopathy, and argyrophilic grain disease. The presence of intraneuronal aggregates of tau best correlates with the neuronal cell death that is associated with the clinical signs and symptoms of disease (*2*). The mechanism of cell death in tauopathy remains unclear; however, several studies implicate microglia in non–cell-autonomous routes (*3*–*5*).

Microglia are the resident immune cells of the brain and, in neurodegeneration, become pathologically activated, leading to proliferation and release of cytotoxic cytokines. Microglia have high phagocytic potential and remove synapses during developmental pruning and disease via phosphatidylserine (PtdSer) (*6*). Recent evidence shows that microglia are also capable of aberrantly phagocytosing synapses and viable neurons in the adult mouse brain (*7*–*9*), and synapse loss is increased in AD (*10*). We have previously shown that inflammation is present in human mutant P301S tau mice (P301S tau) (*11*) and that live cultured dorsal root ganglion neurons (DRGn) from P301S tau mice display PtdSer on the external leaflet of their plasma membranes, which recruits cocultured microglia that phagocytose them while still viable (*9*). This process is accompanied by secretion of the opsonin milk fat globule epidermal growth factor 8 (MFGE8) and nitric oxide production and leads to the transfer of tau aggregates inside the phagocytosed neurons to the microglia (*9*). Microglia have also been shown to phagocytose extracellular tau and become activated to phagocytose neurons in a protein kinase C–dependent manner (*4*). These data implicate microglia in the pathological loss of neurons and synapses in tauopathy by intensifying phagocytic activity to damaging levels.

Tau misfolds into distinct fibrillar forms depending on the specific tauopathy (*12*). Different filamentous forms of tau can template specific pathological conformations onto naïve monomers through a mechanism of prion-like spreading (*13*). Analysis of Braak stage progression suggests that misfolded tau is released as seeds through synaptic connections because anatomically connected regions progressively develop pathology (*14*). Microglia have also been implicated in the spreading of tau pathology (*15*) by releasing exosomes containing tau (*15*, *16*). If microglia release tau seeds after phagocytosing either a tau aggregate–containing neuron or an extracellular tau released at a synapse, they could also act as vectors of tau spreading in the local environment.

Here, we report that in vitro microglia that have phagocytosed living neurons with insoluble filamentous tau aggregates contain tau foci and release insoluble tau aggregates into the conditioned medium (CM). Tau released from such microglia seeds new tau aggregates that are hyperphosphorylated in a reporter system consisting of human embryonic kidney (HEK)–P301S-venus–expressing cells. Microglia cocultured with tau aggregate–containing neurons become hypophagocytic toward both latex beads and a second exposure to tau aggregate–containing neurons that expose the phagocytic signal PtdSer. Evidence for a hypophagocytic microglial phenotype in the brains of P301S mice was confirmed in ex vivo cerebral slice cultures incubated with latex beads. The microglia, recultured following coculture with P301S tau aggregate–containing neurons, also show an increase in senescence-associated β-galactosidase (SA-β-gal) activity. Furthermore, microglia cocultured with aggregated P301S tau-containing neurons secrete a unique signature of proteins that differs from that of lipopolysaccharide (LPS)–stimulated inflammatory microglia. Among these secreted proteins, the active form of matrix metalloprotease 3 (MMP3) is not only highly induced in microglia by coculture with tau aggregate–containing neurons but also increased in the brains of P301S tau transgenic mice and in brains of patients with tauopathies. Consistent with the paracrine signaling properties of the senescence-associated secretory phenotype (SASP), CM from cocultures of microglia and tau aggregate–bearing neurons increased SA-β-gal in naïve, monocultured microglia. Hence, microglia that have phagocytosed neurons containing tau aggregates enter a unique hypofunctional state that resembles aspects of senescence, including the capacity to induce local cellular dysfunction by paracrine signaling with soluble effectors.

## RESULTS

### Microglia that have phagocytosed P301S tau neurons containing tau aggregates release tau

**Fig. 1.**
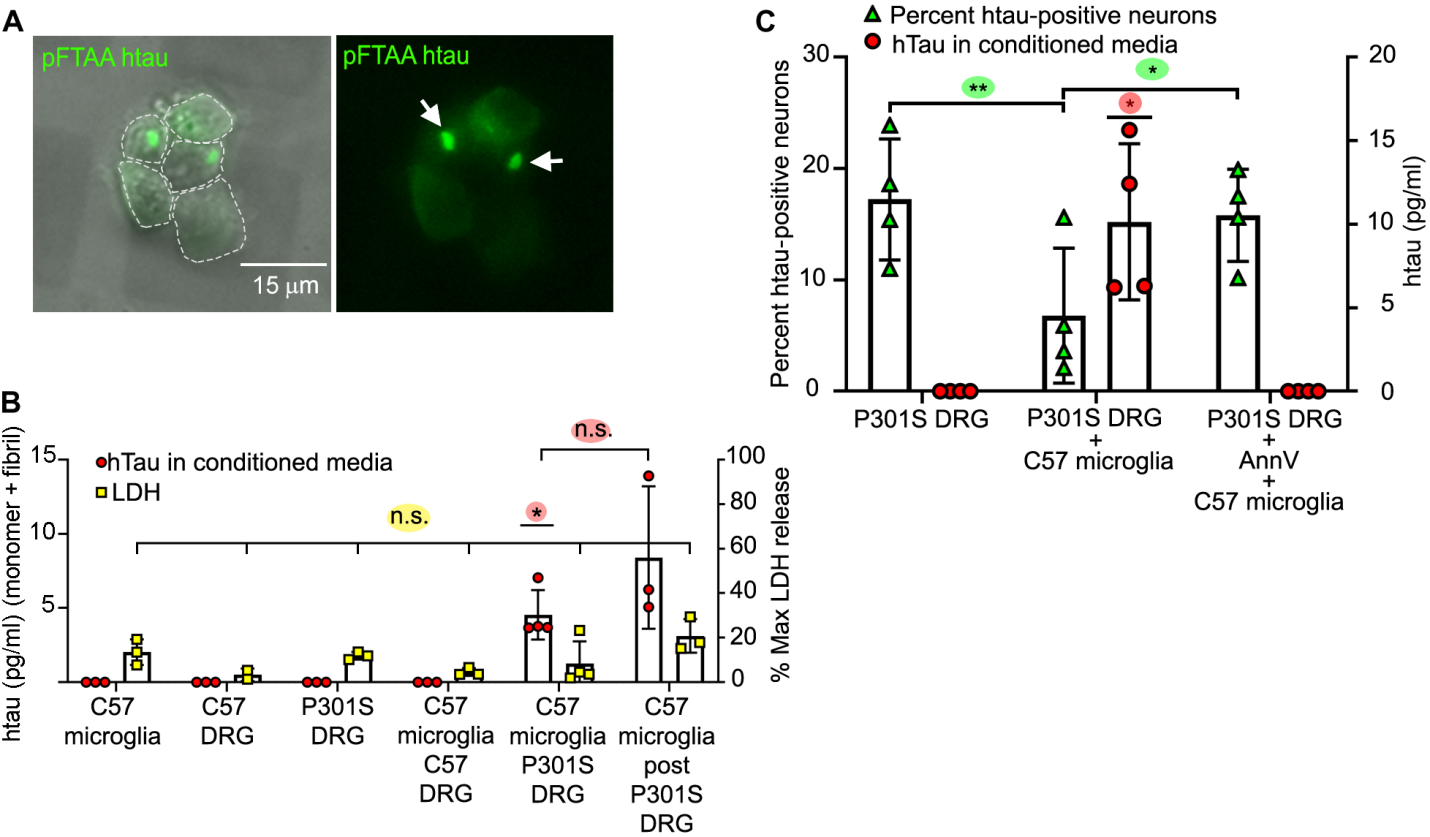
Microglia that have phagocytosed P301S DRGn containing tau aggregates release htau into the CM. (**A**) Tau aggregates in 5M P301S DRGn prelabeled with pFTAA are transferred to microglia after phagocytosis. Microglia were purified after 4 days of coculture, replated, and imaged live. Left: Fluorescence and phase. Right: Fluorescence only. Arrows, pFTAA-positive tau foci. (**B**) htau is only released into the CM when 5M P301S DRGn and microglia are cocultured. Human transgenic tau was detected specifically by ELISA after 4 days (*P* = 0.012, versus all monocultures and coculture of microglia with 5M C57 neurons, one-sample *t* test). Tau is released over a further 4 days by microglia reisolated from 5M P301S DRGn cocultures (post) (*P* = 0.185 versus original coculture CM, unpaired *t* test). No tau is detected in CM from monocultures of 5M P301S or C57 DRGn or C57 microglia or from 5M C57 DRGn-microglia cocultures. Lactate dehydrogenase (LDH) activity is not significantly different across all conditions, indicating that released tau is not due to lysed cells (*P* = 0.069, one-way ANOVA). *N* = 3 to 4 independent experiments, means ± SD. (**C**) Release of tau requires phagocytosis by microglia. The number of htau-positive 5M P301S DRGn (% HT7/βIII-tubulin) is reduced after coculture with C57 microglia (green triangles, *P* = 0.0048, repeated-measures ANOVA), commensurate with an increase in tau in the CM (red circles, *P* = 0.0225, one-sample *t* test). Blocking phagocytosis by masking PtdSer with AnnV prevents neuronal loss (green triangles, n.s. versus P301S DRG, *P* = 0.0309 versus C57 microglia, repeated-measures ANOVA). AnnV also prevents the release of tau into the CM (red circles, n.s. versus P301S DRG). *N* = 4 independent experiments, means ± SD.

DRGn cultures prepared from 5-month-old P301S tau (5M P301S) mice (*17*, *18*) were cocultured for 4 days with wild-type microglia obtained from neonatal C57Bl6J mice (C57) to induce phagocytosis of live neurons containing filamentous tau aggregates as described previously (*9*). Neuronal tau aggregates that were prelabeled with pFTAA before addition of microglia (*19*) were visible as foci of 3 to 5 μm diameter and smaller puncta (Fig. 1A). CMs were assayed for human tau (htau) by enzyme-linked immunosorbent assay (ELISA) after 4 days from either monocultured neurons or microglia, from microglia cocultured with neurons, or from microglia reisolated after coculture (designated as “post”) and cultured alone for a further 4 days (Fig. 1B). No measurable tau was detected in CM from monocultured 5M P301S DRGn containing tau aggregates, C57 wild-type DRGn, or naïve microglia. However, tau (4.54 ± 0.49 pg/ml) was detected in the CM from 5M P301S DRGn-microglia cocultures (average ± SD, *P* = 0.012, one-sample *t* test) and continued to be present even when microglia were reisolated from the cocultures and cultured alone for a further 4 days (post) (8.4 ± 1.3 pg/ml, *P* = 0.185 compared to 5M P301S DRGn-microglia cocultures unpaired *t* test), suggesting that the source of tau was specifically microglia that had phagocytosed neurons with tau aggregates (Fig. 1B). No significant differences in lactate dehydrogenase (LDH) release were detected in the CM across all conditions, demonstrating that tau was not released due to cell death and lysis [*P* = 0.069, one-way analysis of variance (ANOVA) across all conditions] (Fig. 1B).

To further confirm that microglia were the source of tau in the CM post-phagocytosis, PtdSer exposure on neurons was masked with annexin V (AnnV) before coculture (*9*). In the absence of AnnV, the proportion of P301S DRGn neurons containing htau (detected by the HT7 antibody) (% HT7 positive/βIII-tubulin–positive neurons) was significantly reduced ~2-fold when cocultured with microglia (*P* = 0.0048, repeated-measures ANOVA), but this loss was prevented by addition of AnnV (*P* = 0.0309 versus without AnnV, *P* = 0.378 versus naïve) (Fig. 1C). In keeping with a requirement for the engulfment of P301S DRGn for tau release by microglia, tau (10 ± 4.8 pg/ml) was detected in the CM of cocultured 5M P301S DRGn and microglia, but no tau was detected in the CM from cocultures when phagocytosis and loss of tau-positive neurons were prevented by adding AnnV (*P* = 0.0225 comparing ± AnnV, one-sample *t* test) (Fig. 1C). Thus, microglia that have phagocytosed tau aggregate–bearing DRGn are the source of tau released into the CM.

### Tau in the CM is insoluble and seeds tau aggregation

**Fig. 2.**
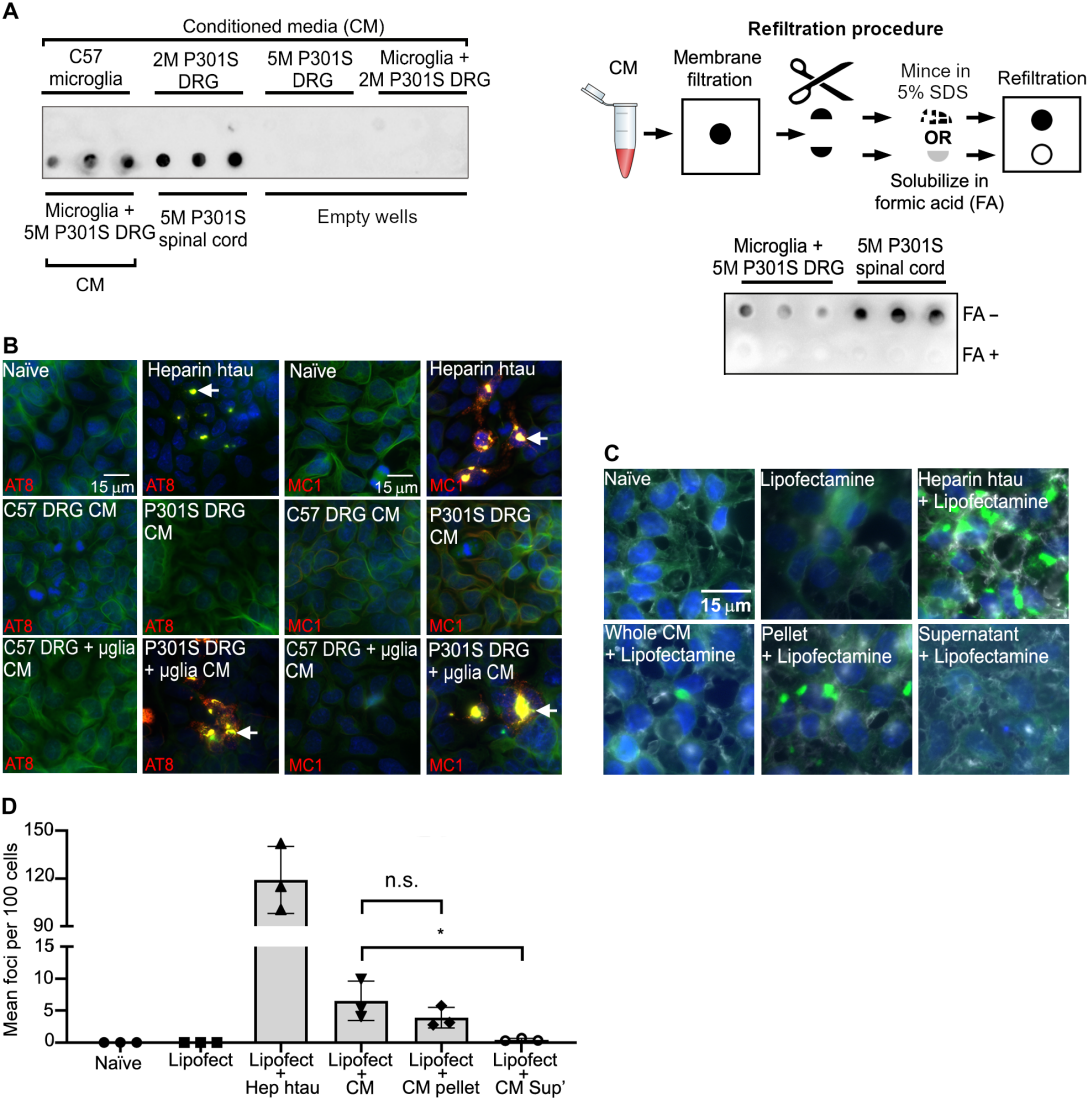
Microglia cocultured with 5M P301S DRGn release insoluble tau aggregates capable of seeding. (**A**) Insoluble tau in the CM is captured on a filter trap (detected with the anti–htau antibody HT7). Top left filter, top row: No tau in CM from monocultures of DRGn (5M C57 and 2M P301S, which do not contain tau aggregates, and 5M P301S) or cocultures of C57 microglia with 2M P301S DRGn. Top left filter, bottom row: htau in CM from cocultures of microglia with 5M P301S DRGn, with spinal cord extracts as positive controls. Bottom right filter: Refiltration of captured tau solubilized in formic acid (FA+) leads to loss of signal, but minced samples (FA−) retain signal. A scheme of the refiltration procedure is also shown (top right). (**B**) Tau in CM seeds new aggregates in the reporter system HEK-P301S-venus cells. The tau-seeded aggregates contain hyperphosphorylated and conformationally altered tau as shown by AT8 and MC1 antibody staining (red). Alexa Fluor-647–conjugated phalloidin (green) was used to outline the cell perimeter. Nuclei were stained by DAPI (blue). (**C** and **D**) Aggregated P301S-venus foci are obtained with the 100,000*g* pellet but not the supernatant of CM after ultracentrifugation (*P* = 0.0429, Friedman test). Representative images and quantification of fractionated CM seeded aggregation in HEK-P301S-venus cells. Lipofectamine was added to increase the efficiency of the uptake. The number of aggregates per 100 cells is quantified in (D). *N* = 3 independent cultures of neurons used for CM preparations, means ± SD.

To investigate whether released tau is monomeric or remains as larger-order aggregates similar to those found in the neurons from 5M P301S mice (*17*, *19*), tau in the CM was solubilized with SDS (5%), and the preparation was filtered through a 0.2-μm cellulose acetate membrane (presoaked in 5% SDS to eliminate soluble oligomers) under vacuum, a method that traps insoluble tau aggregates from AD brains (*20*). Sarkosyl-insoluble tau aggregates from the spinal cord of 5M P301S mice (*9*, *18*) were used as a positive control. CM from cocultured 5M P301S DRGn and microglia contained tau species large enough to be captured on the membrane (Fig. 2A), but no tau was detected in CM from monocultures of microglia, 5M P301S DRGn, or 2M P301S DRGn [which do not contain aggregated tau (*17*)] or from cocultured 2M P301S DRGn and microglia. To further demonstrate that the tau coming from the coculture CM and trapped on the filter was insoluble, each individual filtrate was cut in half as illustrated in the scheme labeled refiltration process in Fig. 2A: One-half was treated with 70% formic acid (FA), which solubilizes tau aggregates (*19*), dialyzed to remove FA, and refiltered. Extracts from the untreated half of the filtrate, which was only minced and soaked in 5% SDS, were also refiltered. FA-treated samples showed no signal, whereas the extracts from the minced samples were still trapped on the new membrane (Fig. 2A), indicating that the tau assemblies trapped on the membrane are insoluble aggregates.

To assay whether tau released from microglia into the CM has aggregation-inducing properties, we used the HEK-P301S-venus cell reporter system (*21*). HEK-P301S-venus cells were incubated with CM for 24 hours, fixed, and immunostained with AT8 (anti–phospho-serine-202/205 antibody) to detect hyperphosphorylated tau or with MC1, which is a conformational antibody that solely detects misfolded tau (*9*, *22*, *23*). Addition of heparin-tau aggregates was used as a positive control. CM from cocultured 5M P301S DRGn and microglia seeded AT8- and MC1-positive aggregates (“naïve” indicates untreated microglia; Fig. 2B), whereas CM from monocultures of C57 or P301S DRGn or 5M C57 DRGn cocultured with microglia showed no seeding capability. To further determine the characteristics of the tau seeds released into CM, the CM was ultracentrifuged at 100,000*g* for 30 min to separate soluble oligomeric species from insoluble aggregates. The pellet was resuspended to the original volume of the CM, and both fractions were added to HEK-P301S-venus cells for 4 days in the presence of Lipofectamine to enhance seed uptake (*21*). The pellet fraction retained a similar aggregation activity to that of whole CM (*P* = 0.662, Friedman test), whereas the supernatant produced significantly fewer foci (*P* = 0.0429, Friedman test) (Fig. 2C, quantified in D). Thus, microglia that have phagocytosed neurons with tau aggregates release tau as species that are competent to seed insoluble tau aggregates in recipient cells.

### Reisolated microglia from P301S tau neuron cocultures are hypophagocytic

**Fig. 3.**
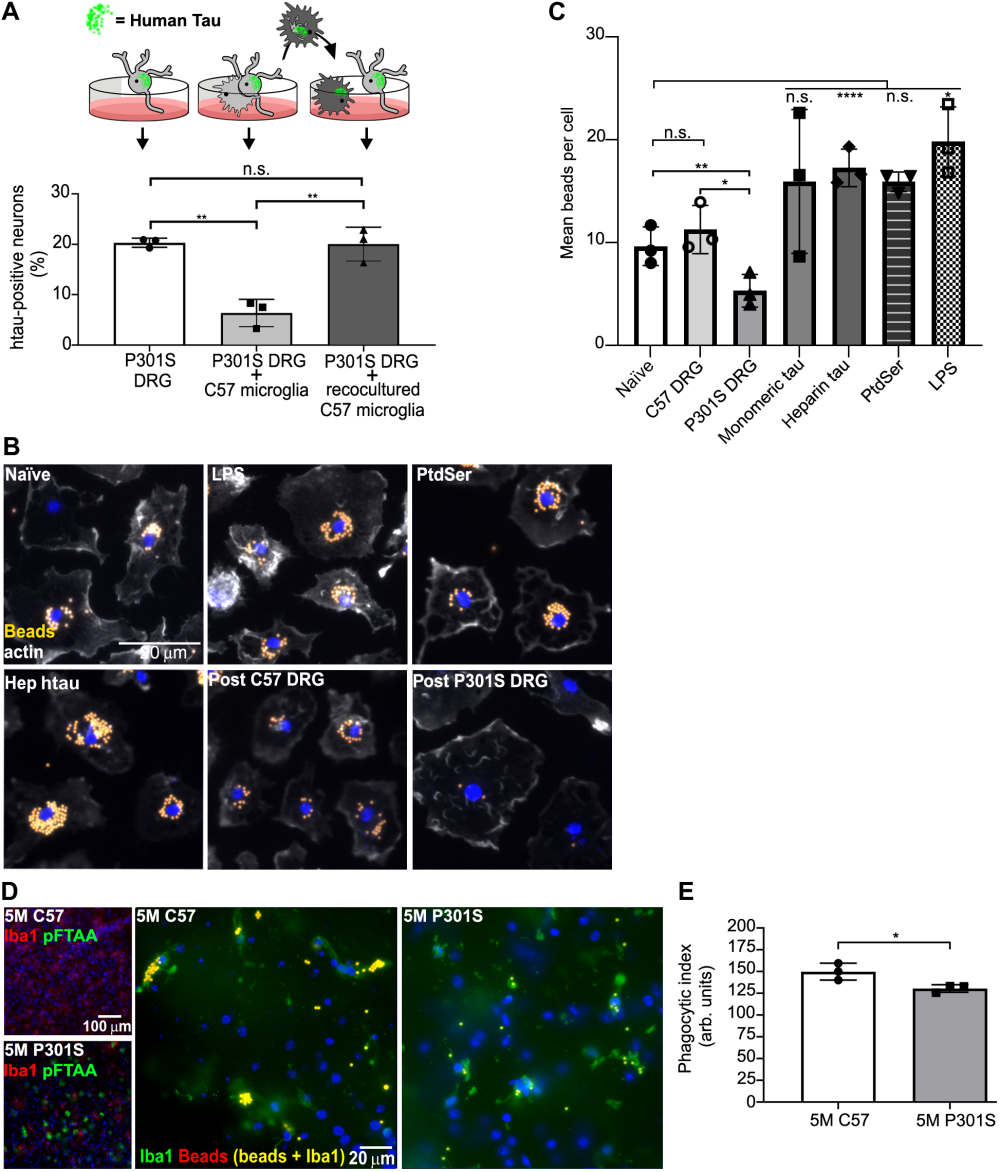
Microglia reisolated from 5M P301S DRGn cocultures or those in 5M P301S brain slices are hypophagocytic. (**A**) Naïve C57 microglia (concave in cartoon) phagocytose P301S DRGn, whereas microglia reisolated from 5M P301S DRGn-microglia cocultures (sunburst shape) are not phagocytic toward a second culture of 5M P301S DRGn (5M P301S DRGn alone versus P301S DRGn with naïve C57 microglia, *P* = 0.0013, versus P301S DRGn reisolated C57 microglia, *P* = 0.9903, one-way ANOVA). *N* = 3 independent P301S DRGn cultures for the first or second cocultures, means ± SD. (**B** and **C**) Microglia reisolated from cocultures with 5M P301S DRGn show a significant reduction of latex bead uptake compared to naïve (untreated) microglia (naïve, *P* = 0.007) or microglia reisolated from coculture with 5M C57 DRGn (*P* = 0.0239, repeated-measures one-way ANOVA); treatment of naïve microglia with PtdSer liposomes does not reduce bead uptake (PtdSer, n.s.), while treatment with heparin-assembled tau significantly stimulates bead uptake (Hep htau, *P* < 0.0001). Quantification is shown in (C). *N* = 3 independent preparations of neurons or microglia exposed to the treatments are indicated, means ± SD. (**D** and **E**) Brain slices (130 μm) from 5M C57 or 5M P301S mice were exposed to latex beads for 3 hours, fixed, and stained for microglia (Iba1, green) to quantify the number of ingested beads (D) (large panels). Matching slices were stained with pFTAA to demonstrate tau aggregates (D) (small panels). Microglia in sections from brains of 5M P301S mice have a significantly reduced phagocytic capacity compared to 5M C57 mice (*P* = 0.0404, Mann-Whitney *U* test). The phagocytic index is displayed in (E). *N* = 3 independent experiments per genotype, means ± SD.

We next investigated whether and how phagocytosis of neurons with tau aggregates affects microglial function. To test their phagocytic capacity, microglia were reisolated from cocultures with 5M P301S DRGn and were transferred to a new monoculture of 5M P301S DRGn. While coculture with naïve, noncocultured C57 microglia caused a significant loss of tau neurons (*P* = 0.0013, one-way ANOVA), no significant loss of tau aggregate–containing neurons was detectable in coculture of reisolated C57 microglia (*P* = 0.9903, one-way ANOVA) (Fig. 3A) (representative images of HT7- and βIII-tubulin–immunostained neurons from 5M P301S before and after coculture with microglia are shown in fig. S1). The loss of phagocytic capacity was unlikely to be due to the changed culture conditions between first and second exposure as microglia isolated from coculture with 5M C57 DRGn and added to 5M P301S DRGn did not show a decrease in phagocytic capacity (25.7 ± 5.8% reduction in the number of P301S DRGn with htau upon exposure to naïve microglia, 18.5 ± 4.5% reduction after adding microglia reisolated from cocultures with 5M C57 DRGn, means ± range of two independent 5M P301S DRGn preparations). These results thus indicate that following ingestion of DRGn containing tau aggregates, the microglia become hypophagocytic and unable to ingest other DRGn with tau aggregates.

Phagocytosis of 5M P301S DRGn is dependent on the specific exposure of the phagocytic signal PtdSer on their membranes (*9*). To investigate whether the inhibition of microglial phagocytosis due to exposure to living P301S DRGn was an independent property acquired by the microglia, reisolated microglia were incubated with carboxylate-modified latex beads. Similar to the suppressed phagocytosis after exposure to 5M P301S DRGn, microglia reisolated from 5M P301S DRGn-microglia cocultures phagocytosed significantly fewer beads than microglia reisolated from 5M C57 DRGn-microglia cocultures (*P* = 0.0239, one-way ANOVA with repeated measures) or nontreated naïve microglia (*P* = 0.007), which had equivalent bead uptake to the microglia reisolated from 5M C57 DRGn-microglia cocultures (*P* = 0.1507) (Fig. 3B, quantified in C). In contrast to the suppression of phagocytosis induced by the coculture of microglia with 5M P301S DRGn, exposure to heparin-tau aggregates increased bead phagocytosis significantly (*P* ≤ 0.0001), suggesting that it was the ingestion of the neurons containing tau aggregates, and not the exposure to aggregated tau, that causes this hypofunctionality. Exposure to monomeric tau also appeared to increase average bead uptake, but the increase was not significant (n.s.) (*P* = 0.5487). Exposure of monocultured microglia to PtdSer liposomes, to mimic the PtdSer exposure on 5M P301S DRGn, caused a similar bead uptake to naïve, monocultured microglia (*P* = 0.557) and was significantly higher compared to the uptake by microglia that had been reisolated from 5M P301S DRGn-microglia cocultures (*P* = 0.0139). These results indicate that the hypophagocytosis by microglia reisolated from 5M P301S DRGn-microglia cocultures was not related to PtdSer signaling. LPS, which was added as a positive control for the induction of microglial phagocytosis, increased bead uptake by the microglia by about twofold compared to the uptake by naïve, monocultured microglia (*P* = 0.028), similar to the uptake induced by monomeric and heparin-tau. Thus, PtdSer exposure is necessary for the phagocytosis of 5M P301S DRGn by microglia but is not sufficient to cause microglia to become hypophagocytic.

### Microglia in cerebral slices from P301S mice are hypophagocytic toward latex beads

To determine whether the hypophagocytic phenotype observed in our coculture systems occurred in vivo in the brain, sagittal slice cultures from 5M C57 and P301S mice were incubated with latex beads using a method previously described (*24*). Abundant pFTAA staining was observed within the cortex of the 5M P301S mice, which was absent in the same brain region in 5M C57 mice (Fig. 3D, small panels). Over a 3-hour incubation period, microglia within the slice cultures phagocytosed latex beads present in the media, as determined by fluorescence colocalization of Iba1 and latex beads. Similar to our in vitro findings, microglia in the cortex of 5M P301S mice, where pFTAA-positive tau aggregates are found, phagocytosed significantly fewer latex beads than microglia in 5M C57 mice (Fig. 3D, large panels; fig. S2A shows a maximum projection confocal image). Results are quantified in Fig. 3E (*P* = 0.0404, Mann-Whitney *U* test). Thus, in the brain, microglia within the same region of aggregated tau–bearing neurons are hypophagocytic toward latex beads.

### Hypophagocytic microglia activate senescence-associated acidic β-galactosidase

**Fig. 4.**
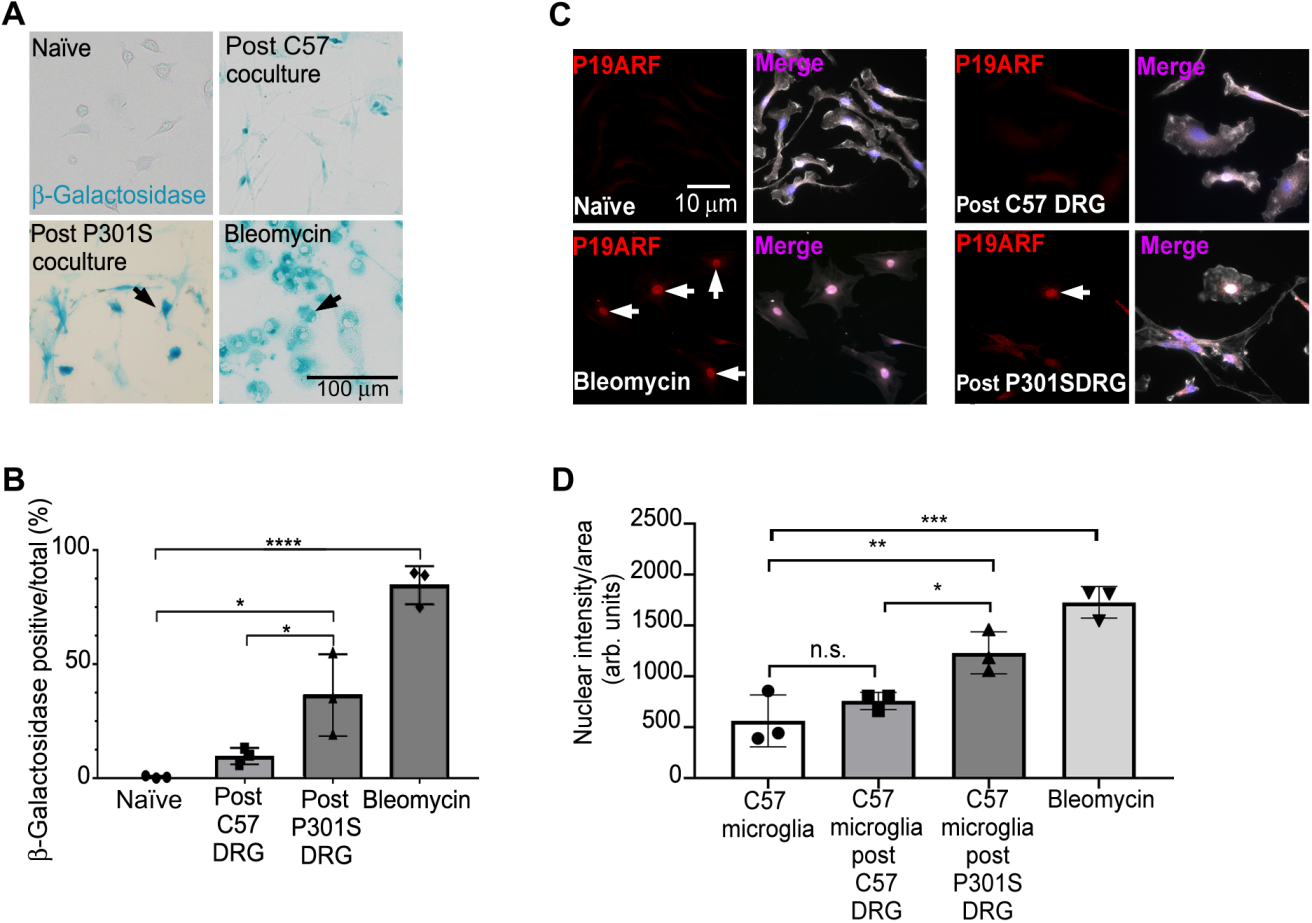
Microglia reisolated from 5M P301S DRGn cocultures display increased SA-β-gal activity. (**A** and **B**) The senescence marker SA-β-gal is significantly increased in microglia reisolated from cocultures with 5M P301S DRGn compared to microglia reisolated from cocultures with 5M C57 DRGn (post P301S DRGn versus post C57 DRGn, *P* = 0.0168) or naïve, monocultured microglia (naïve, *P* = 0.0025, one-way ANOVA and Sidak post hoc). The senescence inducer bleomycin is used as a positive control. Quantification of β-gal is shown in (B). *N* = 3 independent cultures of neurons or microglia, means ± SD. (**C** and **D**) The senescence marker P19ARF (red) undergoes significant nuclear translocation (arrows) in microglia (white; lectin IB4) following coculture with 5M P301S DRGn compared to naïve, monocultured microglia (*P* = 0.0098) and microglia cocultured with 5M C57 DRGn (*P* = 0.0482, one-way ANOVA and Sidak post hoc). The senescence inducer bleomycin is used as a positive control. Nuclear intensity data are quantified in (D). *N* = 3 independent experiments, means ± SD.

Since we had observed a loss of microglial function due to coculture with tau aggregate–containing neurons and pathological tau has been associated with deleterious microglial cellular senescence in a mouse model of tauopathy and in AD (*25*, *26*), we investigated whether senescence was induced in these hypofunctional microglia. We first tested whether microglia reisolated from coculture with 5M P301S DRGn exhibited SA-β-gal activity. There were significantly more β-galactosidase–positive microglia in cultures reisolated from 5M P301S DRGn cocultures compared to microglia reisolated from cocultures with 5M C57 DRGn (~3-fold, *P* = 0.0168, one-way ANOVA) or naïve, monocultured microglia (*P* = 0.0025) (Fig. 4A, quantified in B). As a positive control for the assay, we exposed naïve microglia to bleomycin, which is a well-established inducer of cellular senescence (*27*). Bleomycin (50 μg/ml, 12 hours) up-regulated acidic β-galactosidase activity about eightfold compared to microglia cocultured with 5M C57 DRGn. The presence of elevated β-galactosidase in vivo was confirmed in that an increase in the overall intensity of SA-β-gal staining was observed in cortical sections from 5M P301S mice compared to sections from the same regions of 5M C57 control mice (fig. S2B). The existence of a senescent-like phenotype was further supported by the significant presence of nuclear p19ARF immunostaining in microglia reisolated from coculture with 5M P301S DRGn but not in microglia reisolated from cocultures with 5M C57 DRGn (Fig. 4C, quantified in D). Thus, microglia that have phagocytosed or have been cocultured with 5M P301S DRGn with tau aggregates not only are hypophagocytic but also appear to have acquired a senescence-like state.

### Hypophagocytic microglia release a specific senescence-like protein profile highly enriched in MMP3

To investigate whether microglia that have phagocytosed 5M P301S DRGn produce signals that may account for their hypofunctional post-phagocytic state, we screened the CM for the abundance of 111 mouse proteins (consisting of cytokines, chemokines, growth factors, and matrix-modifying proteases) across multiple treatment conditions using the membrane-based Proteome Profiler Cytokine array (representative images of results from membranes of each condition are shown in fig. S3A, and fig. S3B shows quantification of relative intensities normalized to internal standards across the entire membrane). DRGn monocultures from 5M C57 mice or 5M P301S mice produced no detectable cytokines, but naïve microglial monocultures produced a basal secretory profile that includes CCL2, CCL6, Chemerin, IGFBP-6, mouse colony-stimulating factor 1 (mCSF1), Osteopontin, Serpin E1, and vascular endothelial growth factor (VEGF). LPS treatment of naïve microglia, used to profile an inflammatory phenotype, induced a classical inflammation–associated profile including high levels of tumor necrosis factor–α (TNFα) secretion. In contrast, no proinflammatory cytokines such as TNFα were detected in CM from cocultures of microglia with 5M P301S DRGn. Instead, a different profile of proteins was released.

**Fig. 5.**
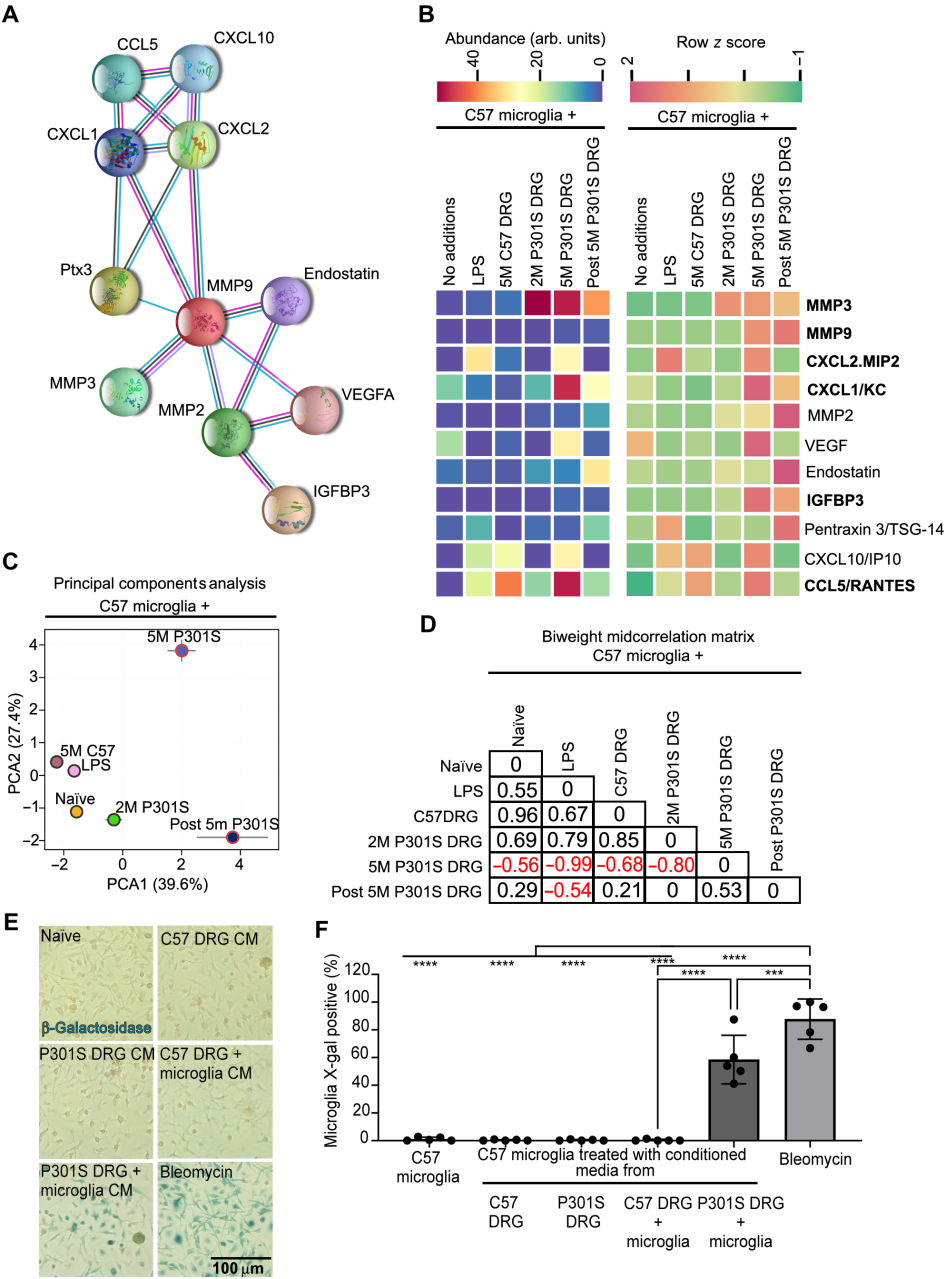
Microglia cocultured with 5M P301S DRGn secrete a unique protein profile. (**A**) Functional interaction network between the 11 proteins identified by STRING analysis. (**B**) Comparative heatmap analysis of protein expression of a family of proteins that are highly enriched in CM from 5M P301S DRGn-microglia cocultures or CM from microglia reisolated from cocultures with 5M P301S DRGn, but not in naïve, monocultured microglia or those cocultured 5M C57 DRGn or 2M P301S DRGn (with no filamentous tau aggregates). Previously published SASP members are in bold. (**C**) PCA plot of proteins presented in (A) (means ± SD, *N* = 3 independent experiments). (**D**) Biweight midcorrelation matrix of the PCA data showing a strong negative correlation between the condition of coculture of microglia with 5M P301S DRGn and all other experimental conditions except for microglia reisolated from cocultures with the 5M P301S DRGn. (**E** and **F**) Microglia treated with conditioned medium (CM) from cocultured 5M P301S DRGn and microglia significantly activate the senescence-associated marker β-galactosidase activity compared to monocultures or microglia cocultured with 5M C57 DRGn (*P* = 1 × 10^−5^ versus naïve, C57 DRG, or P301S DRG, one-way ANOVA with Bonferroni post hoc correction). Data are quantified in (F). Bleomycin is used as a positive control. *N* = 5 independent experiments, means ± SD.

Submitting the full array of proteins to STRING analysis (*28*) identified a network of MMP3 and 14 putative partners (MMP3, MMP9, CXCL2/MIP2, CXCL1/KC, MMP2, VEGF, Endostatin, IGFBP3, Pentraxin2, CXCL10, CCL5/Rantes, CXCL5, CXCL9, CXCL13, and CCL20) (Fig. 5A). Of these, CXCL5, CXCL9, CXCL13, and CCL20 showed no secretion across any of the conditions assayed and were therefore excluded from further analysis. The relative abundance values and row *z* scores for the remaining 11 proteins are shown in Fig. 5B. Six of the proteins listed in bold have been previously associated with the SASP profile (*29*). Principal components analysis (PCA) of the 11 proteins showed that the subset of proteins released by microglia cocultured with 5M P301S DRGn formed a unique group, distinct from LPS-treated microglia or those cocultured with C57 DRGn or 2M P301S DRGn (Fig. 5C). This was further corroborated by the biweight midcorrelation analysis (Fig. 5D).

To examine whether phagocytosis capacity and SA-β-gal are related to ingestion of neurons with tau aggregates or to other signals that arise in the CM as a result of phagocytosis, we exposed naïve microglia to CM isolated from cocultures of 5M P301S or 5M C57 DRGn with microglia or monocultures of these neurons (Fig. 5E, quantified in F). CM from cocultured 5M P301S DRGn and microglia induced a significant number of β-galactosidase–positive microglia, but very few positive microglia were detected under any other culture condition. Bleomycin induced β-galactosidase in 87.7 ± 14.6% of the microglia. CM from cocultures of 5M P301S DRGn and microglia induced β-galactosidase in 58.6 ± 17.6%, *P* < 0.0001 against 5M P301S or 5M C57 DRGn monocultures or cocultured 5M C57 neurons and microglia (*N* = 5 independent preparations of CM, means ± SD, one-way ANOVA with Bonferroni post hoc correction). It remains to be defined whether this effect is induced in microglia due to ingestion of tau-containing neurons and/or their dissolution or to other proteins coingested with tau that were released into the medium. Regardless, this effect of microglia-released proteins is in keeping with the SASP that we observe.

### MMP3 is up-regulated in the CM of cocultured 5M P301S DRGn and microglia and brains of P301S mice and human tauopathies

**Fig. 6.**
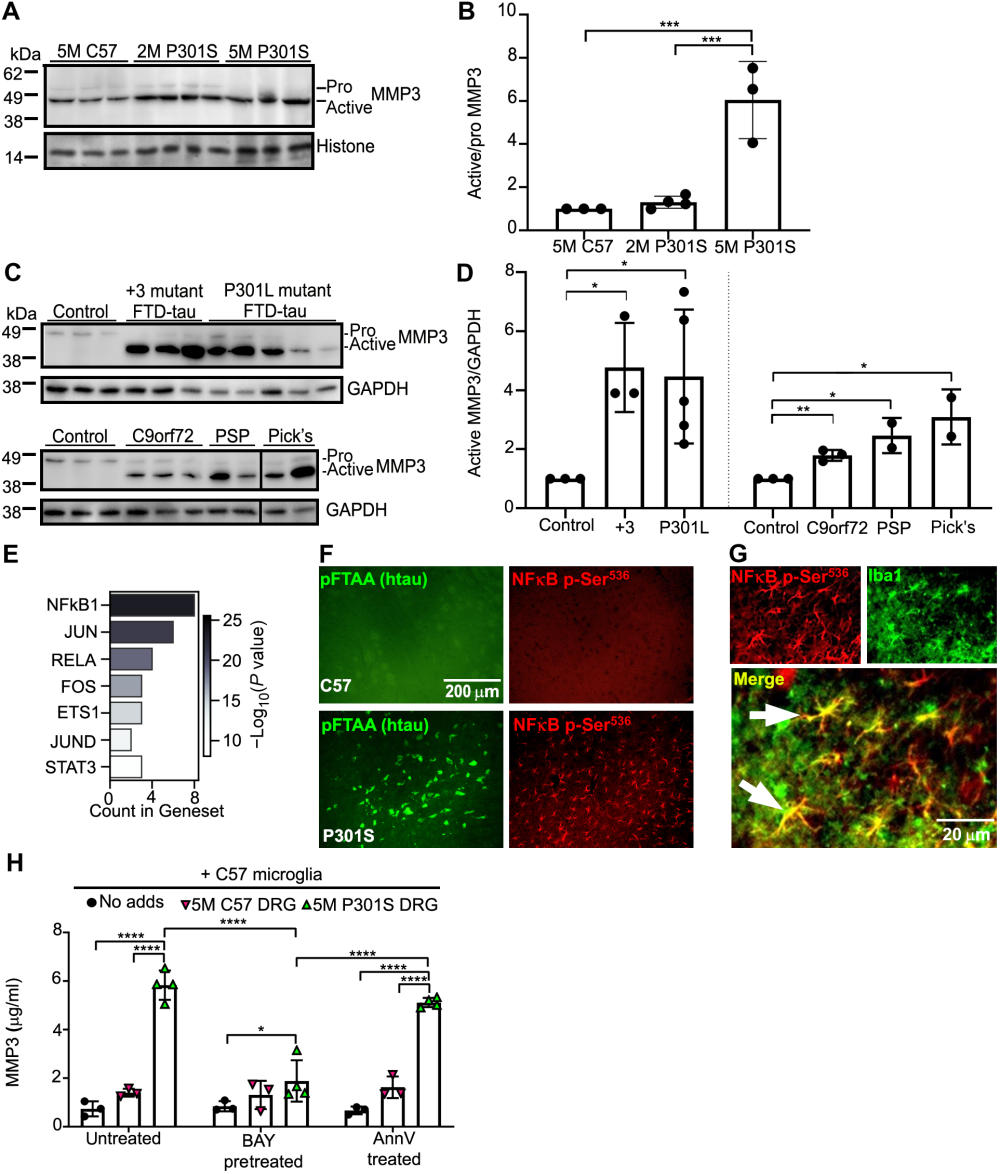
MMP3 is converted to its active form in transgenic P301S mice and human neurodegenerative disease and is under transcriptional control of NFκB. (**A**) Immunoblot of pro and active MMP3 expression in the frontal cortex of 5M C57 wild-type and 2M and 5M P301S transgenic mouse brains. (**B**) Ratio of active/pro forms of MMP3 showing that significantly more active MMP3 is expressed in the brains of 5M P301S mice (5M C57 versus 5M P301S *P* = 0.001, 2M P301S versus 5M P301S, *P* = 0.001; one-way ANOVA). *N* = 3 to 4 independent experiments, means ± SD. (**C**) MMP3 expression in the frontal cortex of controls, +3 and P301L mutant FTDP-17T cases, FTD with C9orf72 mutation cases, Pick’s disease, and midbrain from PSP cases. (**D**) Ratio of active MMP3/GAPDH intensities showing greater active MMP3 expression in neurodegenerative disease versus healthy control (Control versus +3 FTD, *P* = 0.0125; Control versus P301L, *P* = 0.0428; Control versus C9orf72, *P* = 0.0023; Control versus PSP, *P* = 0.0187; Control versus Pick’s, *P* = 0.0244; Student’s *t* test per blot). *N* = 3 to 4 independent experiments, means ± SD. (**E**) The number of proteins identified in the transcriptional regulation network using the Enrichr program; the shaded bar denotes −log_10_ transformation of adjusted *P* values that were computed by Fisher’s exact test within Enrichr. (**F** and **G**) NFκB phosphorylated at Ser^536^ (red) is increased in P301S mouse brainstem (F) (pFTAA, green) and enriched in microglia as shown by colocalization with Iba1 (Iba, green, colocalization in yellow). (**H**) Treatment of microglia with the NFκB pathway inhibitor BAY11-8072 significantly prevents the expression of MMP3 in cocultures of P301S aggregate-containing neurons and microglia. BAY11-8072 was added for 24 hours. AnnV masking of Ptdser over the same period does not prevent MMP3 production (*P* = 10^−5^, two-way ANOVA and Tukey post hoc), means ± SD.

Because MMP3 was prominently up-regulated under the 5M P301S DRGn-microglia coculture condition and was attributed with one of the highest weight coefficients for PC1 (determined by the loading plot analysis presented in fig. S3C), we examined whether its abundance was also increased in mouse and human brains associated with tauopathies. A study of MMP3 expression in the frontal cortex of 5M C57, 2M P301S, and 5M P301S mice by immunoblotting showed two bands corresponding to the inactive zymogen (pro form) and active (mature) MMP3 (*30*). Both the pro and active forms were present in wild-type mouse brain, but there was a highly significant conversion from the inactive to the active form in 5M P301S mice (*P* < 0.001, one-way ANOVA) (Fig. 6, A and B). In the 2M P301S mouse brain, there was no significant increase (*P* = 0.9120) (Fig. 6B). To investigate whether this up-regulation of MMP3 was present in human disease, tissue lysates from the frontal cortex of nondemented controls and patients with Frontotemporal dementia and Parkinsonism linked to chromosome 17 (FTDP-17T) with +3 or P301L MAPT mutations, or Pick’s disease, or from the midbrain of patients with PSP were immunoblotted for MMP3 (Fig. 6, C and D). Frontotemporal dementia (FTD) with TDP-43 pathology due to a C9orf72 expansion mutation was included to investigate whether MMP3 up-regulation is specific to pure tauopathies. The amount of active MMP3 was barely detected in extracts from control brains, but there was a substantial increase in the active/mature form in all the samples from neurodegenerative diseases: FTDP-17T with the +3 and the P301L mutations were elevated 4.7-fold (*P* = 0.013) and 4.5-fold (*P* = 0.043), respectively (ANOVA *P* = 0.04, two-tailed *t* test compared to control), while the ratios were slightly lower for C9orf72 (1.78-fold, *P* = 0.003), PSP (2.45-fold, *P* = 0.019), and Pick’s disease (3.07-fold, *P* = 0.025) (ANOVA *P* = 0.012, two-tailed *t* test compared to control) (Fig. 6D). Hence, active MMP3 is a candidate marker of advanced tauopathies and at least one related form of dementia.

### NFκB regulates MMP3 expression

Previous studies have implicated the nuclear factor κB (NFκB) signaling pathway in the regulation of several of the proteins that we had identified in the CM from 5M P301S DRGn-microglia cocultures (*31*). Submission of the 11 proteins shown in Fig. 5B to the open-source gene set enrichment analysis database Enrichr (*32*) showed that 8 of these proteins, including MMP3, were predicted to be under the transcriptional control of NFκB1, and 4 proteins were predicted to be under the transcriptional control of RelA (NFκB p65 subunit) (Fig. 6E). We performed staining of brain sections from 5M C57 and P301S mice with an anti–phospho-Ser^536^-NFκB antibody to determine whether NFκB signaling could be involved in the behavior of microglia in the mice. Abundant staining for phospho-Ser^536^-NFκB that colocalized with microglia (detected by Iba1 staining) was observed in brain sections from P301S mice, whereas few microglia showed staining above background in sections from C57 mice (Fig. 6, F and G). To further investigate whether NFκB controls the production of MMP3 in our experimental paradigm, microglia were preincubated with the NFκB pathway inhibitor BAY11-8072 (*33*) for 1 hour, after which they were cocultured with 5M C57 or 5M P301S tau DRGn. CM was collected after 24 hours, and MMP3 was measured by ELISA. BAY11-8072 significantly reduced the amount of MMP3 in the CM from 5M P301S DRGn-microglia cocultures (*P* < 0.0001, two-way ANOVA with Tukey post hoc) (Fig. 6H). BAY11-8072 induced no change in the amount of MMP3 detected in 5M C57 DRGn-microglia cocultures. To investigate whether MMP3 secretion induced by 5M P301S tau DRGn in microglia was due to phagocytosis of the neurons, phagocytosis was prevented by preincubating the neurons with AnnV. Unlike BAY11-8072, AnnV blockade of phagocytosis did not reduce the production of MMP3 (n.s. compared to no 5M P301S DRG additions), indicating that phagocytosis-related hypofunctionality and NFκB-dependent MMP3 production are activated by different effectors emanating from the 5M P301S DRGn. These results show that while hypofunctionality is a consequence of the physical act of phagocytosis linked to PtdSer, MMP3 activation appears to depend on additional signals that are PtdSer-independent, in keeping with the induction of SA-β-gal obtained when microglia were exposed to CM from cocultures of microglia with 5M P301S DRGn (Fig. 5, E and F).

## DISCUSSION

**Fig. 7.**
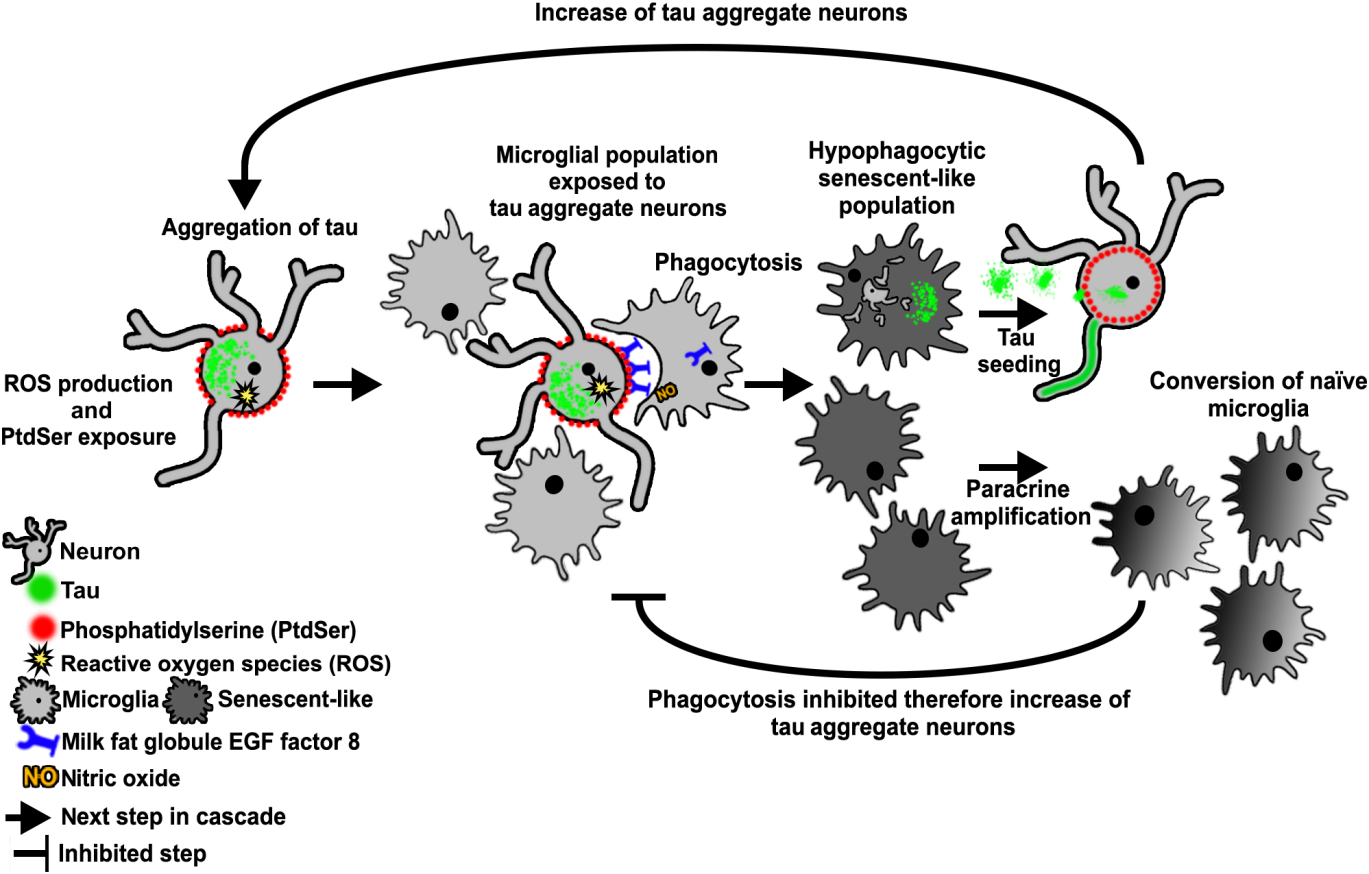
Schematic of proposed microglial dysfunction associated with phagocytosis of tau aggregate–containing neurons. Tau aggregate–containing neurons increase reactive oxygen species (ROS) production and expose PtdSer, which recruits cocultured microglia to perform phagocytosis. When phagocytosis occurs, the community of microglia undergoes phenotypic transitions characterized by hypophagocytic behavior and the acquisition of senescence-associated traits. Microglia that have phagocytosed tau aggregate–containing neurons secrete seeding-competent tau aggregates that can induce further tau aggregation, while paracrine factors can convert naïve microglia to become hypophagocytic/senescent-like. The end result is an increasing number of neurons with tau aggregates in a self-amplifying cycle.

Inflammation and microgliosis are a common feature of many neurodegenerative diseases, and microglial phagocytosis is suggested to be highly important in disease pathogenesis with the discovery of phagocytosis genes as genetic risk factors in AD (*34*). Here, we report that after phagocytosis of neurons with P301S tau aggregates, microglia release forms of tau that can seed new tau aggregates. On a population level, microglia become hypophagocytic, and they display several elements of senescence, including senescence-associated acidic β-galactosidase activity and release of senescence-associated proteins, some of which are under NFκB transcriptional control. It remains to be resolved whether the expression of senescent markers, the onset of a hypophagocytic phenotype, and the capacity for paracrine-mediated dysfunction are responses unique to microglia that have ingested tau aggregate–containing neurons or whether other microglia within the coculture undergo these phenotypic transitions independently of direct phagocytosis. In the latter case, the self-amplification of tau aggregation and microglial dysfunction could be extensive. A scheme illustrating how these events might lead to a vicious cycle of neurodegeneration is shown in Fig. 7.

Our data show that microglia that have phagocytosed neurons with insoluble tau aggregates not only maintain these aggregates for several days but also expel aggregates capable of seeding tau aggregation. Ingestion of neurons with tau aggregates was necessary for tau to appear in the medium since AnnV prevented phagocytosis of P301S neurons and also prevented tau release in cocultures of P301S DRGn and microglia, so tau release is unlikely to be a result of neuronal cell death induced outside the microglia or other factors in the culture environment. Our data showing an extended period of tau release are in agreement with a study where microglia isolated from adult rTg4510 mouse brains or AD brains also released tau over several days that was capable of seeding tau aggregation in a fluorescence resonance energy transfer–based biosensing cell assay (*35*). In the same study, inflammatory stimuli failed to increase the release of tau seeds from microglia, and LPS failed to induce the same secretome profile as that released from microglia cocultured with 5M P301S DRGn, so perhaps, microglia with senescent phenotypes similar to those that we demonstrate here are those most prone to release tau seeds.

Microglia have previously been implicated as vectors of tau spreading in vivo through exosomes in mouse models with focal viral delivery of tau–green fluorescent protein (*16*). However, no filamentous or misfolded tau was detected in these experiments, so it remains to be shown whether tau would be released by microglia via exosomes if it were highly aggregated, as is the case here. Although tau aggregate pathology is thought to develop along synaptically connected routes through neuronally released tau seeds (*14*, *36*), evidence for lateral spread of tau has been reported (*37*). Thus, it is possible that microglia surrounding neurons with tau pathology could act as sources of tau seeds, especially after PtdSer-dependent phagocytosis of tau aggregate–containing neurons or synapses that display PtdSer (*6*). It is interesting that the presence of filamentous aggregated tau inside microglia in human tauopathy cases is a rare event (*38*), despite well-described cases of tau pathology in astrocytes and oligodendrocytes (*39*). It is possible that microglia have a more efficient means of degrading or expelling tau aggregates (*40*) than astrocytes or oligodendrocytes. Another possibility is that microglia with tau aggregates fragment and die due to acquisition of a senescent phenotype (*26*), thereby releasing tau seeds. However, there was no noticeable microglial death in our cocultures based on sequential micrographs taken over the 4-day coculture period or in the study of Hopp *et al.* (*35*), so the mechanism of tau aggregate release remains to be identified.

The present data demonstrate that microglia show hormetic behavior: Having been activated to engulf neurons with tau aggregates, the community of microglia become hypophagocytic and show signs of senescence. Exposure to tau per se is unlikely to be the cause of the functional sequelae because of the following: (i) Blocking phagocytosis with AnnV also blocked the release of tau seeds (Fig. 1C); (ii) acute exposure to tau/tau seeds increased bead uptake (Fig. 2C); (iii) Pampuscenko *et al.* (*4*) showed that addition of tau to cocultures of wild-type neurons and microglia increased phagocytosis of the living neurons (via PtdSer exposure) as well as increased phagocytosis of beads in pure microglial cultures; and (iv) we do not find an effect equivalent to that of 5M P301S neurons using neurons from 2M P301S mice, which have not developed aggregates and are not engulfed (*9*). Thus, it is possible that the engulfment of a minority of neurons that contain tau aggregates is sufficient for the population-level hormetic effect that we observe.

The precise mechanism by which microglia become hormetic remains to be resolved. We tested whether PtdSer could be the signal since phagocytosis of live neurons by microglia depends on PtdSer exposure on the neuronal membrane (*4*, *9*, *41*, *42*), but hormesis was not mimicked by exposure of microglia to PtdSer liposomes, nor was MMP3 secretion reduced by masking PtdSer with AnnV. Moreover, acidic β-galactosidase activity in C57 microglia only occurred after exposure to CM from cocultured 5M P301S DRGn with microglia. Given that acute exposure to tau monomers or aggregates causes an increase in phagocytosis, it seems likely that the appearance of hormesis is mediated by other factors released into the medium as a result of ingestion of neurons with tau aggregates or other factors released due to exposure of microglia to tau aggregate–containing neurons, consistent with the SASP profile that we observe.

The phenomenon of microglial hormesis after phagocytosis of β-amyloid has been described previously. For example, microglia that have phagocytosed β-amyloid fibrils showed reduced phagocytosis toward β-amyloid oligomers (*43*). Hypophagocytic behavior was also observed in vivo in two mouse models with β-amyloid plaques (*24*) and demonstrated by the failure of freshly isolated microglia from 5xFAD transgenic mice to clear β-amyloid plaques from organotypic 5xFAD slice cultures, whereas wild-type microglia displayed ample clearance (*44*). Our data also support the contention that microglia in vivo in 5M P301S mice are also hypophagocytic. The switch from a pro-resolution phenotype to a hypophagocytic phenotype was suggested to represent a proinflammatory state that occurs when the microglia fail to digest and degrade engulfed, highly stable filamentous protein aggregates (*45*, *46*). However, we did not observe a hypophagocytic microglial phenotype when microglia were fed with tau aggregates. The molecular differences underpinning the different states of the microglia under these different hormetic settings remain to be clarified.

In addition to microglia being hypophagocytic, we show that coculture of microglia with P301S tau aggregate–containing neurons induced the microglia to secrete several senescence-associated factors [CXCL1, CXCL2, CCL5, IGFBP3, MMP9, and MMP3 (*29*)]. The presence of dystrophic microglia that have been linked to senescence has been reported in AD and other neurodegenerative disorders (*47*–*50*), although no molecular signatures of senescence were recorded. Loss of phagocytic capacity, increased SA-β-gal activity, induction of senescence-associated genes such as CDKN2A/P19ARF, and altered cytokine profiles such as those we describe here are common signs of senescence. In this hypofunctional state, microglia may lose their neuroprotective role and possibly exacerbate neurodegeneration. The mechanism of transduction to senescence is unclear, although both cytokine signaling and phagocytic stress have been suggested to induce senescence in retinal epithelium (*51*). It will be important to identify the biochemical mechanism of these changes and determine whether modification of the process would be beneficial in disease.

The production of MMP3 was one of the most notable changes induced by coculture of microglia with 5M P301S neurons. A recent transcriptomic analysis (*52*) describes an anti-inflammatory microglial subtype that is induced by inflammation, but this may not resemble the response that we observe as none of the proteins that we have identified in the CM are present as altered transcripts aside from CXCL10 and CCL2, both of which are linked to an inflammasome interferon signaling module. A weak link to phagocytosis is the up-regulation of ARF and CDC42 transcripts, which modify the dynamics of actin polymerization, but with these being more active, one may expect more pronounced phagocytosis rather than an inhibition such as the one that we observe. Nevertheless, activation of MMP3 is a hallmark of several tauopathies, including AD and familial FTD cases (*53*, *54*). Since NFκB has been implicated in control of MMP3 expression in other cell types (*31*, *55*), it was of interest to examine to what extent NFκB-dependent MMP3 production is linked to tau release from cocultured microglia. We found that the NFκB inhibitor BAY11-7082 prevented MMP3 production despite being applied under culture conditions that allow phagocytosis of the neurons and therefore release of tau by microglia. An involvement of NFκB was corroborated by the presence of phospho-Ser^536^-NFκB in microglia in 5M P301S mouse brain sections. MMP3 was still highly produced in the presence of AnnV, however, which blocks phagocytosis. Thus, NFκB appears to be activated as a result of cell signaling either between neurons with tau aggregates and microglia or between post-phagocytosis microglia and surrounding microglia. This would also account for the increase in SA-β-gal–positive microglia following exposure to CM from cocultures of microglia with P301S DRGn. Since TNFα was not induced by the neurons, another possible mediator of NFκB activation is the reactive oxygen species produced by neurons with tau aggregates (*19*, *56*). The consequence of such strong up-regulation of an MMP remains to be resolved. MMP3 could have effects at the synapse due to its tissue remodeling properties via the extracellular matrix and/or by altering the chemokine secretome (*57*–*59*).

The key message from our study is that microglia switch to an alternative, active functional state that is not classically inflammatory as a result of coculture of neurons containing tau aggregates. Microglia that have phagocytosed tau aggregate–containing neurons produce tau seeds, which may enhance tau pathology not only by spreading tau aggregation to new neurons but also by contributing to loss of normal phagocytic functions throughout the community of microglia.

## MATERIALS AND METHODS

### Experimental design

The objective of these experiments was to test the functional state of microglia after phagocytosis of neurons containing insoluble filamentous hyperphosphorylated tau aggregates and to determine the fate of the ingested tau. The study was designed as an in vitro cell culture system to investigate cellular and biochemical mechanisms leading to phenotypic changes in microglia and consequences for pathology, confirming key findings using brains from transgenic human P301S tau mice and human brain tissue from neurodegenerative diseases. We have previously published that DRGn from P301S mice with tau aggregates expose PtdSer and are phagocytosed live by cocultured microglia (*9*).

### Mice

Homozygous mice transgenic for human 0N4R P301S tau (*18*) (henceforth P301S) and C57BL/6S (C57BL/6OlaHsd; henceforth C57) control mice were maintained as described previously (*17*). P301S tau mice were aged to 5 months, when phenotype is evident. Each independent experiment (*N*) was derived from an independent culture from an individual mouse. This research project was performed under the Animals (Scientific Procedures) Act 1986, Amendment Regulations 2012 following ethical review by the University of Cambridge Animal Welfare and Ethical Review Body. Both female and male mice were used for experiments. Mice were housed in groups in individually ventilated cages, adding a plastic roll and nesting material for enrichment. Mice were kept under a 12-hour light/dark cycle, with food and water available ad libitum.

### Human tissue

Human tissue was obtained from the Cambridge Brain Bank and the Alzheimer’s Center at Indiana University. Handling of human tissue was according to the U.K. Human Tissue Act 2006 and is covered by the Cambridge Local Research Ethics Committee, approval number 04/090. Each case was neuropathologically confirmed as being either control, Pick’s disease, PSP, or expressing mutant P301L or +3 or C9ORF72 as described previously (*9*). The cohort contained mixed sexes, and the age at death ranged from 53 to 76 (full details in table S1). Fresh-frozen frontal cortex or midbrain was homogenized in radioimmunoprecipitation assay containing 2.5% SDS with protease inhibitors (Roche) at a ratio of 1:2 (w/v) with an IKA T-10 Ultra Turrax. Whole homogenates were clarified by centrifugation at 20,000*g* for 30 min at 4°C. The protein content in the resulting supernatant was determined by bicinchoninic acid (BCA) assay (Pierce), and 5 μg of protein was analyzed by immunoblot.

### Cell cultures

DRGn from either P301S or C57 mice were cultured either on six-well culture plates coated with poly-d-lysine or laminin and maintained as reported previously (*17*). To visualize tau aggregates, neurons were labeled in medium containing 3 μM pFTAA (a gift from K. P. R. Nilsson, Linköping University, Sweden, as previously described). Primary microglial cells were prepared from postnatal day 2 to 4 pups as previously described and cultured in Dulbecco’s modified Eagle’s medium (DMEM) containing 1% Antibiotic-Antimycotic (PSF) (Thermo Fisher Scientific, 15240062) and 2 mM GlutaMAX (Gibco, 35050061) (henceforth DMEM/PSF/Gmax), 10% heat-inactivated fetal bovine serum (FBS; Thermo Fisher Scientific, 10082147), and mCSF1 (50 ng/ml; Peprotech, 315-02). For coculture experiments, 5M P301S or C57 DRGn were cultured for 7 days in DMEM/PSF/Gmax containing 1% heat-inactivated FBS and 20 μM fluorodeoxyuridine to remove non-neuronal cells. After washing the DRGn in phosphate-buffered saline (PBS), 200,000 primary microglia were added directly to the neurons, and mixed cultures were maintained in DMEM/PSF/Gmax and 5% heat-inactivated FBS for 4 days at which time the CM was collected. For transfer experiments, microglia were reisolated from the first coculture by physical dissociation in ice-cold PBS without Mg^2+^ or Ca^2+^, passed through a 40-μm-pore membrane to exclude any contaminating DRGn, and added to a fresh culture of P301S DRGn in the same medium containing 5% heat-inactivated FBS for a further 4 days. For testing bead uptake and SA-β-gal activity, reisolated microglia were plated onto glass coverslips for 4 days in microglial growth medium containing mCSF1 (10 ng/ml). NFκB inhibition in microglia was performed by adding 5 μM BAY11-7082 (Sigma-Aldrich, B5556) for 1 hour before microglial addition to DRGn. AnnV (100 nM) (ImmunoTools, 31490010) was added to the DRGn to pretreat and mask PtdSer, thus preventing phagocytosis.

### Seeding assay

HEK-P301S-venus cells were maintained in DMEM, 10% FBS, and 1% PSF on 13-mm uncoated glass coverslips. To assay the tau aggregate seeding efficiency of CM, maintenance medium was replaced with CM for 48 hours in the absence or presence of Lipofectamine 2000 diluted 1:100 (*21*). Cells were then fixed in 4% paraformaldehyde (PFA) and immunostained as below.

### Immunocytochemistry

Tau aggregates in P301S DRGn were labeled live with pFTAA as described previously (*19*, *23*). To detect and quantify the number of neurons with htau, neurons were costained with anti–htau monoclonal antibody HT7 (1:500; Thermo Fisher Scientific, MN1000) and anti–βIII-tubulin (1:1000; Sigma-Aldrich, T2200). Between 100 and 200 neurons were counted along a strip that extended from the top to the bottom of a 13-mm coverslip using a 10× objective (about 10 fields) in each independent culture for determining the ratio of HT7/βIII-tubulin. Reisolated microglia were counterstained with DAPI (4′,6-diamidino-2-phenylindole) and/or phalloidin-Alexa Fluor-647 (Life Technologies) and then mounted in FluorSave (Millipore). HEK-P301S-venus cells were permeabilized in 0.3% Triton X in PBS (PBST) and incubated with primary antibody overnight in PBST using AT8 antibody (1:1000; Thermo Fisher Scientific, MN1020), to detect hyperphosphorylated tau, and MC1, as described previously (*23*). Cells on coverslips were washed in PBS and incubated with appropriate Alexa Fluor-conjugated secondary antibody for 1 hour at room temperature in PBST, counterstained with DAPI (0.1 μg/ml), and mounted in FluorSave. For staining of brain sections with anti-phospho-Ser^536^ NFκB antibody (1:500; Cell Signaling, 3031S), 25-μm sections of 5M P301S or 5M C57 mouse cortices that had been postfixed in 4% PFA (*19*, *23*) were stained overnight at 4°C with primary antibody in PBST followed by anti-rabbit biotinylated antibody (1:500; Vectorlabs, BA-1000) and streptavidin-conjugated Alexa Fluor-568. To colocalize staining with microglia, sections were stained with goat anti-Iba1 (1:250; Abcam, ab5076) followed by anti-goat Alexa Fluor-647. Sections were then stained with 3 μM pFTAA and DAPI (0.1 μg/ml). Images were taken on a Leica DMI 4000B microscope using a Leica DFC3000 G camera and the Leica application suite 4.0.0.11706. Images were analyzed using ImageJ (Rasband, W.S., ImageJ, U.S. National Institutes of Health, Bethesda, MD, USA; http://imagej.nih.gov/ij/). Micrographs of each fluorescence channel were used to count neurons with ImageJ cell counter plugin. P19ARF/CDKN2 antibody (1:500; Novus, NB200-106) was applied to fixed microglia and visualized using anti-rabbit Alexa Fluor-568. Microglial perimeters were defined using the lectin IB4 conjugated to fluorescein isothiocyanate (Vectorlabs, FL-1201-.5). Nuclear P19ARF intensity was quantified using ImageJ to measure pixel intensity and nuclear area.

### Enzyme-linked immunosorbent assay

DRGn from P301S or C57 mice were cocultured with C57 microglia for 4 days to generate CM where tau was assayed. CM was first centrifuged at 20,000*g* for 20 min to remove any cellular debris, and the supernatant was collected. Tau protein concentration in CM was assayed with anti–htau ELISA according to the manufacturer’s instructions (Abcam, ab210972). LDH assay was performed on the CM according to the manufacturer’s instructions (Abcam, ab102526). To assay the effect of NFκB inhibition, DRGn from P301S or C57 were cocultured with C57 microglia that had been pretreated with BAY11-7082 or left untreated for 24 hours to generate CM for MMP3 assay. MMP3 ELISA (Abcam, ab100731) was performed on CM 24 hours after addition of microglia according to the manufacturer’s instructions.

### Immunoblotting

Cells were lysed in 1% NP-40, 137 mM NaCl, 2 mM EDTA, and 20 mM tris-HCl (pH 8) with protease inhibitor cocktail (Roche, 04693116001) and assayed for protein concentration by the BCA assay (Pierce, 23225). Equal protein amounts were added to Lithium dodecyl sulfate (LDS) loading buffer (Bio Rad 161-0747) containing 10% β-mercaptoethanol and run on 4 to 12% gradient SDS–polyacrylamide gel electrophoresis gels. Proteins were transferred onto 0.2-μm-pore polyvinylidene difluoride membranes (Merck, IPVH00010). Nonspecific background was blocked in 5% (w/v) dry skimmed milk (Sigma-Aldrich) in PBS containing 0.1% Tween 20, and membranes were incubated with the primary antibody (Abcam, ab53015) overnight at 4°C, followed by 1 hour at room temperature in the appropriate horseradish peroxidase–labeled secondary antibody (GE Healthcare). Blots were developed with enhanced chemiluminesence Clarity (Bio-Rad, 1705061). Intensity was quantified using ImageJ. In mouse samples, the ratio of pro to active MMP3 was used to express the percentage of active MMP3, but the ratio of active MMP3 to glyceraldehyde-3-phosphate dehydrogenase (GAPDH) was used for human samples because no pro-MMP3 was detected in some samples.

### Membrane filtration assay

CMs were centrifuged at 20,000*g* for 20 min to remove cellular debris. Cellulose acetate membranes (0.2 μm) were soaked in 5% SDS in dH_2_O for 10 min at room temperature and then mounted onto a 96-well vacuum chamber. Samples were incubated in 5% SDS in dH_2_O for 10 min at room temperature, and 2 ml of each CM was filtered through the membrane under vacuum. Membranes were washed in 5% SDS under vacuum, rinsed in PBS and 0.1% Tween 20, then incubated with primary antibody (HT7; 2 μg/ml) overnight at 4°C, and developed using the method described for immunoblotting. A sarkosyl-insoluble extract from a 5-month-old P301S mouse spinal cord prepared as described previously (*18*) was the positive control. After initial signal development, each membrane dot was cut out of the membrane and bisected; half was treated with 70% FA for 15 min and then dialyzed overnight with Slide-A-Lyzer (Thermo Fisher Scientific, 66203) in PBS and 0.1% Tween 20. The other half was minced, vortexed, and soaked in 5% SDS to elute available captured protein. Processed samples were then membrane-filtered, antibody-incubated, and developed as before.

### Latex bead phagocytosis assays

To assay phagocytic activity in cell culture, fluorescent latex carboxylate-modified polystyrene beads (2.0 μm mean particle size) were added to reisolated microglial cultures on 13-mm coverslips at 1:10,000 dilution for 12 hours at 37°C or added to cultures of matched cohorts pretreated with either LPS (100 ng/ml) for 1 hour, 12 nM monomeric tau, 12 nM heparin-fibrillized tau (monomer equivalent), or PtdSer vesicles (1 μg/ml) prepared by dissolving PtdSer in chloroform and drying under nitrogen before hydration in PBS and sonication to produce a vesicle suspension. Cultures were washed in PBS to remove free beads and fixed in 4% PFA. Microglia were counterstained with DAPI and phalloidin-Alexa Fluor-647 and mounted with FluorSave. Images were collected using the Leica DMI 4000B microscope and analyzed using ImageJ.

To assay the phagocytic activity of microglia in acute brain sections, fresh brains from 5M C57 or P301S mice were immediately submerged in ice-cold *N*-methyl-d-glucosamine (NMDG) artificial cerebrospinal fluid (aCSF) (*60*). The brains were bisected along the midline, and both hemispheres were mounted together (medial surfaces down) on the cutting stage of a Leica VT1200S vibratome with superglue. Sequential 130-μm sections were generated (amplitude: 0.7 μm; speed: 0.7 μm/s). Anatomically matched pairs of slices were collected in a 24-well plate containing 500 μl per well of ice-cold NMDG-aCSF, with one slice per well. Slices were left to recover in the incubator (5% CO_2_, 37°C) for 2 hours, after which 2-μm fluorescent latex beads were added into the medium of one set of slices at a 1:100 dilution and left for 3 hours. The other matching sets of slices were kept without beads. Sections were thoroughly washed in PBS to remove free beads and fixed in 4% PFA. All sections were stained overnight at 4°C with goat anti-Iba1 antibody (1:500, Abcam, ab5076) to assess microglial colocalization. The sections incubated with beads were stained with an Alexa Fluor-488 anti-goat secondary antibody (1:500) in PBST, while the sections without beads were stained with an Alexa Fluor-647 anti-goat secondary antibody (1:500) for 1 hour at room temperature, followed by staining with pFTAA (3 μM). All sections were stained with DAPI (1 μg/ml). Sections were mounted with Fluoromount-G. Images for quantification were collected using a Leica DMI 4000B microscope and analyzed using ImageJ. Phagocytic index was computed as described previously (*24*). Briefly, the number of beads per cell counts was stratified into bins of 1 to 4, 5 to 7, 8 to 10, and >10. Each bin was assigned a corresponding weight (1 to 4:1, 5 to 7:2, 8 to 10:3, and >10:4). For each mouse, the percentage of cells within each bin was multiplied by the weight of the corresponding bin, which was then summed and displayed as the phagocytic index.

### SA-β-gal assay

Reisolated microglia were plated onto 13-mm coverslips and fixed in 4% PFA. The activity of β-galactosidase at pH 6.0 was assayed in a staining solution consisting of 5-bromo-4-chloro-3-indolyl-β-d-galactopyranoside (1 mg/ml; X-gal; Invitrogen, AM9944), 10 mM citrate-sodium phosphate buffer (pH 6.0), 5 mM potassium ferricyanide, 5 mM potassium ferrocyanide, 150 mM NaCl, and 2 mM MgCl_2_ overnight (18 to 20 hours) at 37°C. Bleomycin (50 μg/ml) was added as a positive control inducer of chemical senescence for 12 hours. Coverslips were washed and mounted in FluorSave. For CM treatments, CMs from five independent cocultures of microglia with 5M C57 or 5M P301S neurons or monocultures of the same neuron preparations were added to a single preparation of C57 microglia on coverslips for 4 days. Cultures were fixed, stained, and mounted as per reisolated microglia. For brain sections, 25-μm floating brain sections that had been postfixed in 4% PFA were incubated in the X-gal mixture for 18 hours at 37°C; pFTAA staining was used to verify P301S tau pathology. Colorimetric X-gal staining was imaged using an Olympus BX50 microscope and QCapture Pro 7 QImaging suite with a Retiga 2000R Fast 1394 camera.

### Cytokine array

CM was collected and centrifuged at 20,000*g* to remove cellular debris. Comparative cytokine amounts were assayed with the proteome profiler XL mouse cytokine array as per the manufacturer’s instructions (R&D, ARY028). Densitometric measurements were quantified using ImageJ and normalized between membranes using the membranes’ positive controls. To identify expression pathways, the data were analyzed by STRING analysis [(*28*), https://string-db.org/]. Interactions permitted were experimentally determined, coexpressed, and those that were predicted to be expressed by databases; any noninteracting proteins were removed from the analysis. Selecting the most confident partner interactions in the remaining array (threshold, ≥0.9) yielded the following unique proteins: CXCL9, CXCL10, CXCL5, CXCL2, CXCL1, CCL5, CCL20, ENDOSTATIN, VEGFA, MMP2, CXCL13, IGFBP3, MMP9, PTX3, and MMP3. Cxcl5, Cxcl9, Cxcl13, and Ccl20 were omitted due to lack of detectable secretion across all conditions. The abundance of 11 proteins across three independent biological replicates was expressed as a mean. The raw abundance values were standardized by calculating *z* scores for each protein across all conditions, such that 0 represents the group mean, and each SD from the mean is represented by integer values (e.g., −2, −1, 1, and 2). These results were then displayed on a heatmap.

### Principal components analysis

PCA was conducted using the Scikit-Learn library in Python (version 3.7.6) (*61*). To calculate error bars (SD), full replicate data (*n* = 3) were used to “train” the PCA transformation matrix, and error was calculated for each condition from the PC1 and PC2 coordinates of the replicates. This same transformation matrix was then applied to the mean-only dataset, and the error bars calculated previously were interpolated onto each corresponding condition.

### Statistical analysis

Statistical analysis and graphing were performed using GraphPad Prism version 7. Samples were compared using normal or repeated-measures ANOVA followed by an appropriate post hoc test or by Student’s *t* test as indicated. A one-sample *t* test was used when a mean was compared to a hypothesized mean value of zero because the measurement was below the level of detection. Repeated-measures ANOVA with matched pairs was specifically applied when comparing different culture conditions on the same sample source. Mann-Whitney *U* test was used for the acute brain section bead phagocytosis assay, as the data were not normally distributed (Shapiro-Wilk test). An independent experiment is defined as being sourced from a single animal. Significant differences are reported as *P < 0.05, **P < 0.01, ***P < 0.001, and ****P < 0.0001.

## Supplementary Materials

### This PDF file includes:

Figs. S1 to S3 Table S1 References

## Notes

View/request a protocol for this paper from *Bio-protocol*.
