# Supplementary material for: Microglia become hypofunctional and release metalloproteases and tau seeds when phagocytosing live neurons with P301S tau aggregates
Source: Sci Adv. 2021 Oct 20;7(43):eabg4980. doi: 10.1126/sciadv.abg4980 (PMC8528424; doi:10.1126/sciadv.abg4980)
Supplement: Supplementary file 1 — Figs. S1 to S3 Table S1 References [file sciadv.abg4980_sm.pdf]

Science Advances
AAAS

# Supplementary Materials for

## Microglia become hypofunctional and release metalloproteases and tau seeds when phagocytosing live neurons with P301S tau aggregates

Jack H. Brelstaff, Matthew Mason, Taxiarchis Katsinelos, William A. McEwan, Bernardino Ghetti, Aviva M. Tolkovsky*, Maria Grazia Spillantini*

*Corresponding author. Email: mgs11@cam.ac.uk (M.G.S.); amt1004@cam.ac.uk (A.M.T.)

Published 20 October 2021, *Sci. Adv.* **7**, eabg4980 (2021)
DOI: 10.1126/sciadv.abg4980

**This PDF file includes:**

Figs. S1 to S3
Table S1

Supplementary Figure S1

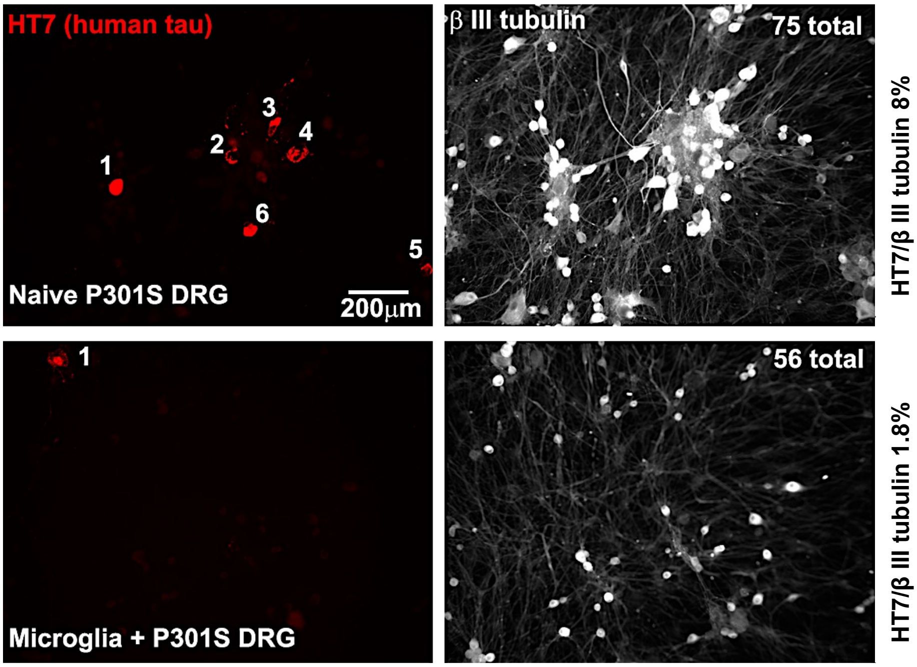


An example of HT7+ve neurons co stained with βIII tubulin from monocultures of 5M P301S DRGn (top panels) and following co-culture with C57 microglia (bottom panels), with reference to Figure 3A.

Supplementary Figure S2

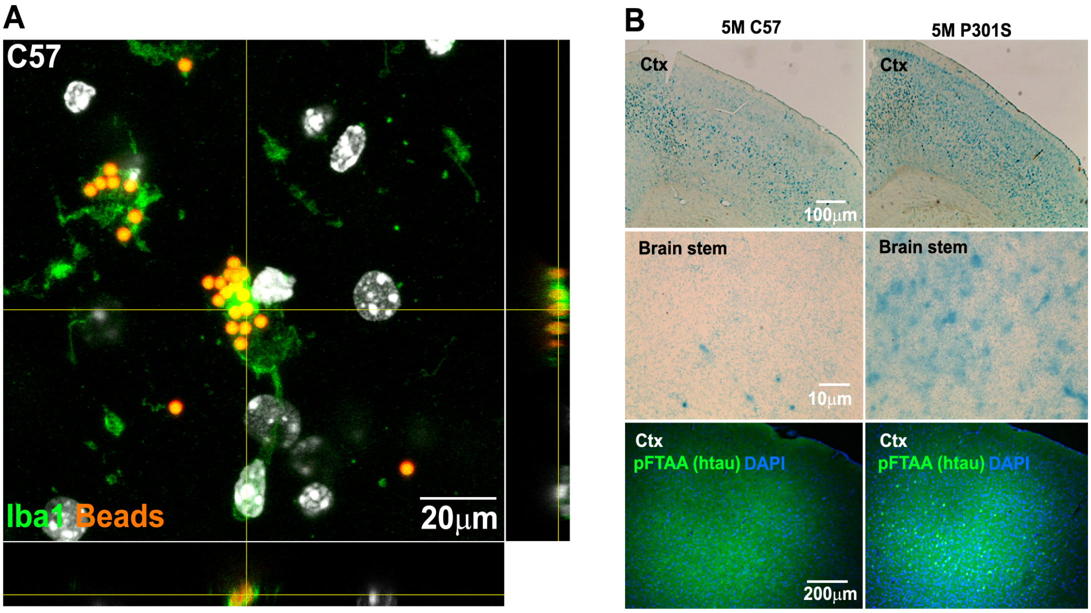


**A)** Maximum projection confocal image of microglia in a brain section from a 5M C57 mouse after incubation with latex beads, with reference to Figure 3D and E. Orthogonal views demonstrate that beads (red) have been internalised by the microglia (Iba1 in green). Nuclei stained with DAPI are shown in white.

**B)** Images of SA-β-gal-stained brain sections from 5M C57 and 5M P301S mice with reference to Figure 4. Cortical and brain stem sections (25 μm) were stained for 12 hours with acidic x-gal solution. Top panels, overview of the cortex (4x objective). Middle panels, brain stem (approximately interaural −2 mm, bregma −5.7 mm) (20x objective). Parallel cortical sections stained with x-gal were post-stained with pFTAA and DAPI under fluorescence to show tau aggregates in 5M P301S mice (10x objective).

Supplementary Figure S3

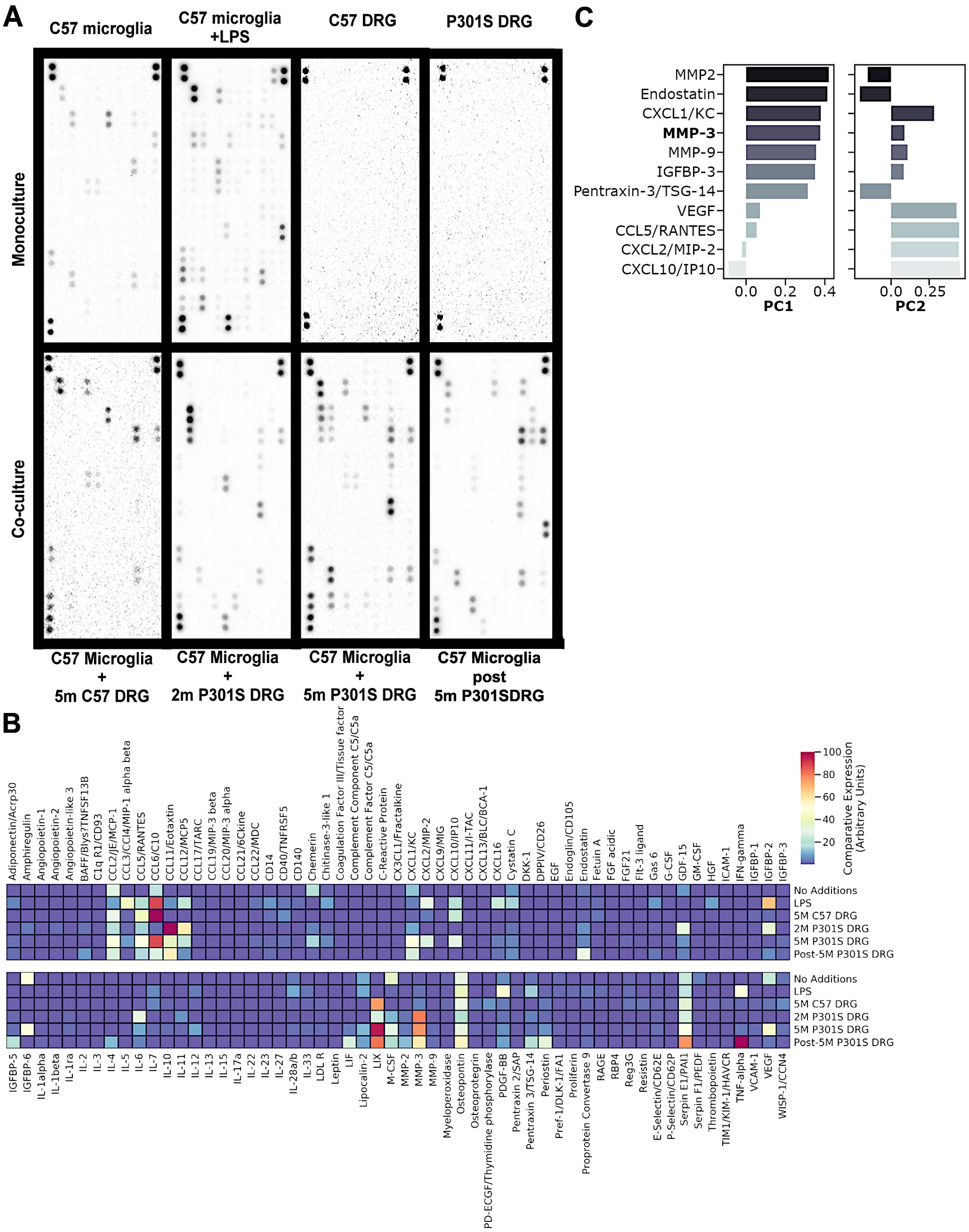


**A)** Examples of one set of membrane arrays with reference to Figure 5. Membranes probed with CM from (left column) monocultures of untreated microglia, microglia treated with LPS, pure DRGn from 5M C57 or P301S mice, and (right column) co-cultures of microglia with DRGn from 5M C57, 2M P301S, or 5M P301S mice, and microglia isolated and recultured from co-cultures with 5M P301S mice, used for quantification The pair of spots on the top left, top right, and bottom left of each membrane are internal positive controls, and the rightmost pair of (invisible) spots on the bottom right are internal negative controls.

**B)** Relative expression heat map of total proteins on the cytokine arrays . The internal positive and negative controls are not represented.

**C)** Loadings plot of the two principal components, showing that MMPs dominate the PC1 axis whereas PC2 is dominated by the cytokines CXCL1, -2, -10, and CCL5.

**Supplementary Table S1. Human tissue details in relation to Figure 6.**

Human brain tissue was obtained from The Alzheimer Disease Center, Indiana University School of Medicine and the Cambridge Brain Bank. Handling of human tissue was according to the UK Human Tissue Act 2006 and is covered by the Cambridge Local Research Ethics Committee (LREC), approval number 09/40. Grey matter (0.2 g) was mechanically homogenized in RIPA buffer containing 2.5% SDS with phosphatase and protease inhibitors at a 1:2 (w/v) ratio. Lysate was clarified at 20,000 g for 30 min and protein assayed.

Abbreviations: FTDP-17T, frontotemporal dementia and Parkinsonism linked to chromosome 17, MSTD, Multiple system tauopathy with presenile dementia, FTLD, frontotemporal lobar degeneration. Controls are defined age matched subjects who did not present neurological disorders.

| Sample order | Disease or mutation | Gender | Age at death | Neuropathology |
|---|---|---|---|---|
| Blot 1 | | | | |
| 1 | Control | F | 56 | Moderate cerebral atherosclerosis |
| 2 | Control | F | 66 | Metastatic adenocarcinoma |
| 3 | Control | M | 65 | Cerebral atrophy |
| 4 | MAPT+3 | F | 58 | FTDP-17T (MSTD) |
| 5 | MAPT+3 | F | 61 | FTDP-17T (MSTD) |
| 6 | MAPT+3 | M | 64 | FTDP-17T (MSTD) |
| 7 | P301L | M | 59 | FTDP-17T |
| 8 | P301L | F | 55 | FTDP-17T |
| 9 | P301L | F | 62 | FTDP-17T |
| 10 | P301L | M | 63 | FTDP-17T |
| 11 | P301L | F | 55 | FTDP-17T |
| Blot 2 | | | | |
| 1 | Control | F | 56 | Moderate cerebral atherosclerosis |
| 2 | Control | M | 65 | Metastatic adenocarcinoma |
| 3 | Control | F | 66 | Cerebral atrophy |
| 4 | C9ORF72 | F | 42 | FTLD |
| 5 | C9ORF72 | M | 69 | Neurodegenerative disease |
| 6 | C9ORF72 | F | 65 | FTLD with TDP-43 type B |
| 7 | PSP | M | 79 | PSP |
| 8 | PSP | M | 67 | PSP |
| 9 | Pick Disease | F | 76 | $Tau^+$ Pick bodies and balloon neurons |
| 10 | Pick Disease | M | 75 | $Tau^+$ Pick bodies and balloon neurons |
